# Architectural evolution in cocoons spun by *Hyalophora* (Lepidoptera; Saturniidae) silk moth species

**DOI:** 10.1038/s41598-020-62547-1

**Published:** 2020-03-27

**Authors:** Patrick A. Guerra, Lucinda P. Lawson, Lea J. Gatto, Molly E. Albright, Scott J. Smith

**Affiliations:** 1 Department of Biological Sciences, University of Cincinnati, Cincinnati, OH USA; 2 9807 E. Broadway Ave., Spokane Valley, WA 99206 USA

**Keywords:** Evolutionary ecology, Entomology, Ecology, Evolution

## Abstract

Caterpillars of the silk moth genus *Hyalophora* (Lepidoptera; Saturniidae) construct multilayered cocoons that function as overwintering housing during the pupal to adult developmental period. While all cocoons share the primary function of protecting the developing moth, cocoons spun by different *Hyalophora* silk moth species vary significantly in architectural features and in the level of intraspecific cocoon-type polymorphism. We compared the cocoons of *Hyalophora* species found throughout North America and investigated the evolution of architectural variation. We first characterized and compared the architectural features of cocoons at all three cocoon sections (outer envelope, inner envelope, and the intermediate section that separates them), and found that variation in the outer envelope underlies the differences in architecture between cocoons. Phylogenetic analysis indicates ancestral polymorphic architecture (both “baggy” and “compact” morphs), with diversification within *Hyalophora* for both monomorphic “compact” cocoons, and increased intermediate space and silk in “baggy” lineages. The evolution of these traits suggests a potential functional role for the different cocoon architectural forms.

## Introduction

Individuals from many diverse taxa have evolved to build structures that house and protect the individual from environmental stress. Many of these structures have been adapted to possess specific architectural features that facilitate their ability to buffer against adverse local environmental conditions^1,2^. As part of an extended phenotype^3^, the architectural characteristics of these structures can also be under selection pressure, creating the potential for both the form and function of these structures to change over time. Differential selection on architectural features can explain the diversity in constructs exhibited even between closely related species.

The cocoons spun by silk moth species in the genus *Hyalophora* (Lepidoptera; Saturniidae) are examples of structures that can protect individuals against adverse environmental conditions^4–6^. Built during the summer by caterpillars in the final fifth instar larval stage, these cocoons protect individuals while they overwinter as pupae. The following spring, individuals emerge from the cocoons as adults.

Cocoon architecture varies within *Hyalophora*, with some species (e.g., *Hyalophora cecropia*) producing discrete dimorphic cocoons of either a large and fluffy cocoon (baggy) or a significantly smaller and tightly woven cocoon (compact)^6,7^. Other species produce cocoons with continuous variation in morphology (*Hyalophora euryalus*^8,9^), and still others produce only the compact form (*Hyalophora columbia*, *Hyalophora* cf *gloveri*, and the proposed hybrid lineage *Hyalophora “kasloensis”* that result from crosses of *H. euryalus* x *H*. cf *gloveri*^8^). As variation within *H. cecropia* is linked to a locale-dependent strategy for dealing with adverse environmental conditions during the pupal stage, the architecture of the cocoons of these other *Hyalophora* species may also play a similar role though local adaptation to range-specific environmental conditions^9^.

**Figure 1:**
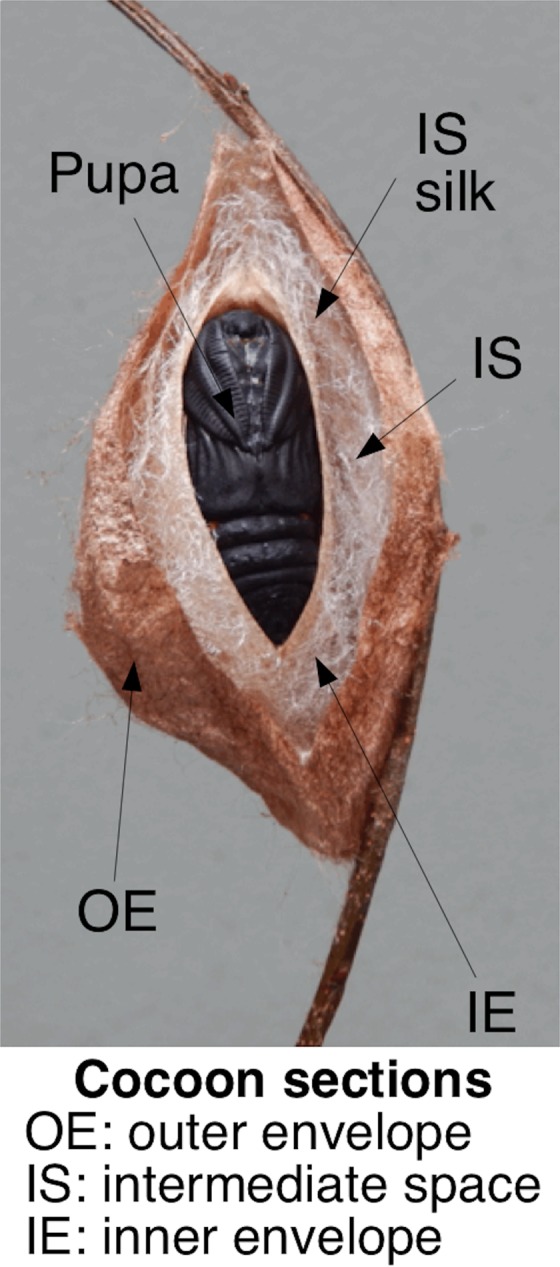
The different architectural sections of cocoons made by *Hyalophora* silk moth species. Photo was taken by Steven M. Reppert and is adapted from Guerra & Reppert^6^.

*Hyalophora* cocoons all have two discrete envelopes (inner and outer; Fig. 1), but vary in the intermediate space, and the presence and abundance of intermediate silk. Cecropia moth caterpillars (*H. cecropia*), for example, produce a multilayered cocoon with an intermediate space between the layers filled with silk^6,10,11^. This intermediate space and silk, combined with two distinct morphs (baggy and compact) appears to mitigate environmental stochasticity during pupal development. The multi-layered and dimorphic architecture of cecropia moth cocoons produces alternative cocoon types with specific biophysical advantages relative to stochastic environmental conditions during development, conditions that can vary over the large environmental gradients encompassed by the entire habitat range of these moths. Population variation in cocoon architecture across the large environmental gradient encompassed by this lineage, indicates that this may be a locale-dependent, bet-hedging strategy^6^. Other *Hyalophora* moths within this genus may have different combinations of these architectural traits and may lack the developmental plasticity to create these kinds of morphs to tailor developmental cocoon conditions to environmental variation.

**Figure 2:**
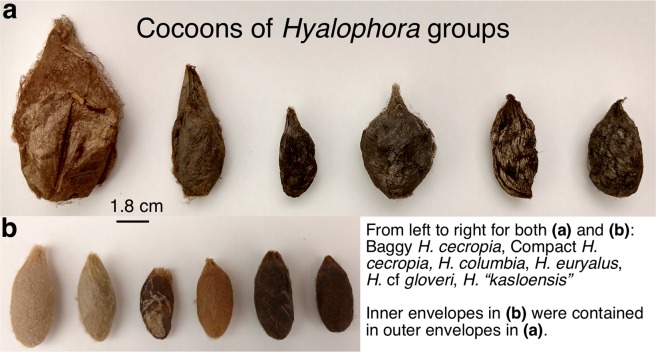
Representative cocoons from the *Hyalophora* species examined in our study. **(a)** Outer envelopes of cocoons. **(b)** Inner envelopes of cocoons. Inner envelopes pictured are the inner envelopes that were contained within the outer envelopes directly above in **(a)**.

In this study, we used the cocoons of different species within *Hyalophora* (*H. cecropia*, *H. columbia*, *H. euryalus*, and *H*. cf *gloveri*) and that of a hybrid (*H. “kasloensis”*), as a model to examine the evolution of architecture and dimorphism in animal construction (Fig. 2a,b). To address this, we first used three-dimensional (3D) analysis to characterize and compare the architectural features (i.e., size and shape) of cocoons across the groups at all three levels of construction: outer envelope (Fig. 2a), inner envelope (Fig. 2b), and intermediate space. We also compared the cocoons with respect to the total amount of silk used for construction and the allocation of silk between the different cocoon sections. Next, we conducted a phylogenetic analysis to determine how different architectural features and the existence of multiple cocoon morphologies have evolved within the genus. Together, these results inform on whether the diverse cocoon architectures in *Hyalophora* are consistent with a strategy for dealing with environmental conditions during the pupal developmental period prior to adult eclosion.

## Results

### Different amounts of silk building materials

A single *H. euryalus* cocoon from our sample contained only an outer envelope, i.e., no inner envelope was spun, but the larval pellet and pupal casing were found inside this otherwise normal (size and shape) outer envelope. This cocoon was therefore omitted from our silk analyses.

We found that the cocoons from the different *Hyalophora* groups contained different amounts of total cocoon silk (One-way ANOVA: F_5, 58_ = 18.7600, p < 0.0001). Post hoc comparisons (Tukey HSD test, ∝ = 0.05) showed that loose/round (*H. euryalus*) and baggy (*H. cecropia*) cocoons had a higher amount of silk than cocoons with compact morphology (p < 0.0001 for all comparisons between *H. euryalus* and compact cocoon groups, and for all comparisons between baggy *H. cecropia* and all compact cocoon groups). *Hyalophora euryalus* and *H. cecropia*, however, were indistinguishable from each other (*H. euryalus*: 0.8726 +/− 0.0537 g, n = 9; *H. cecropia*: 0.7800 +/ 0.0389 g, n = 10). Between the two *H. cecropia* cocoon morphologies, compact cocoons (0.6800 +/ 0.0389 g, n = 10) had similar amounts of total silk as baggy cocoons, but contained significantly less silk than *H. euryalus* cocoons. For the three species with monomorphic compact cocoons, the cocoons of *H*. cf *gloveri* (0.6292 +/ 0.0486 g, n = 10) had intermediate amounts of total silk, and the cocoons with the least amounts of total silk belonged to *H. “kasloensis”* (0.4851 +/ 0.0403 g, n = 10) and *H. columbia* (0.3786 +/ 0.0293, n = 10). *Hyalophora* groups that produce polymorphic cocoons (*H. cecropia* and *H. euryalus*) contain more total silk than groups that produce monomorphic cocoons (p < 0.0001 for all post hoc comparisons between each of *H. cecropia* and *H. euryalus*, with that of *H*. cf *gloveri*, *H. “kasloensis”*, and *H. columbia*, respectively).

We found that cocoons from the different *Hyalophora* groups significantly differed in their percentage of total cocoon silk that was partitioned to the outer envelope (Kruskal-Wallis test: χ^2^ (5) = 45.3088, p < 0.0001). Post hoc Wilcoxon pairwise comparisons (∝ = 0.05) found that although loose and round *H. euryalus* cocoons had a significant percentage of total silk in the outer envelope, overall, groups that spun compact cocoons allocated the greatest proportion of total silk to the outer envelope. Compact *H. “kasloensis”* cocoons were similar to *H. euryalus* cocoons, and also had the greatest percentage of total cocoon silk in the outer envelope. Compact *H. columbia* and compact *H*. cf *gloveri* cocoons had intermediate percentages of total silk devoted to the outer envelope, followed by *H. cecropia* compact cocoons. Baggy *H. cecropia* cocoons had the lowest percentage of total silk devoted to the outer envelope.

Cocoons from the different *Hyalophora* groups significantly differed in their percentage of total cocoon silk found in the intermediate space of cocoons (Kruskal-Wallis test: χ^2^ (5) = 48.5039, p < 0.0001). Post hoc Wilcoxon pairwise comparisons (∝ = 0.05) showed that baggy *H. cecropia* cocoons had the greatest percentage of total silk in the intermediate space, and this percentage was significantly greater than the percentages of all other groups. Compact *H. cecropia* cocoons had the next largest percentage of total silk in the intermediate space, followed by *H. euryalus*, *H*. cf *gloveri*, and *H. “kasloensis”* cocoons, which all had similar percentages of total silk. *Hyalophora columbia* cocoons contained no silk in the intermediate space.

The cocoons from the different *Hyalophora* groups differed in the percentage of total silk found in the inner envelope (Kruskal-Wallis test: χ^2^ (5) = 37.3455, p < 0.0001). This difference was not as marked across groups, however, as this difference was due to a significant difference between two clusters: baggy *H. cecropia*, compact *H. cecropia*, *H. columbia*, and *H*. cf *gloveri* having a significantly greater percentage of total silk in the inner envelope than *H. euryalus* and *H. “kasloensis”* (post hoc Wilcoxon pairwise comparisons, ∝ = 0.05).

### Outer envelopes – size and shape

**Figure 3:**
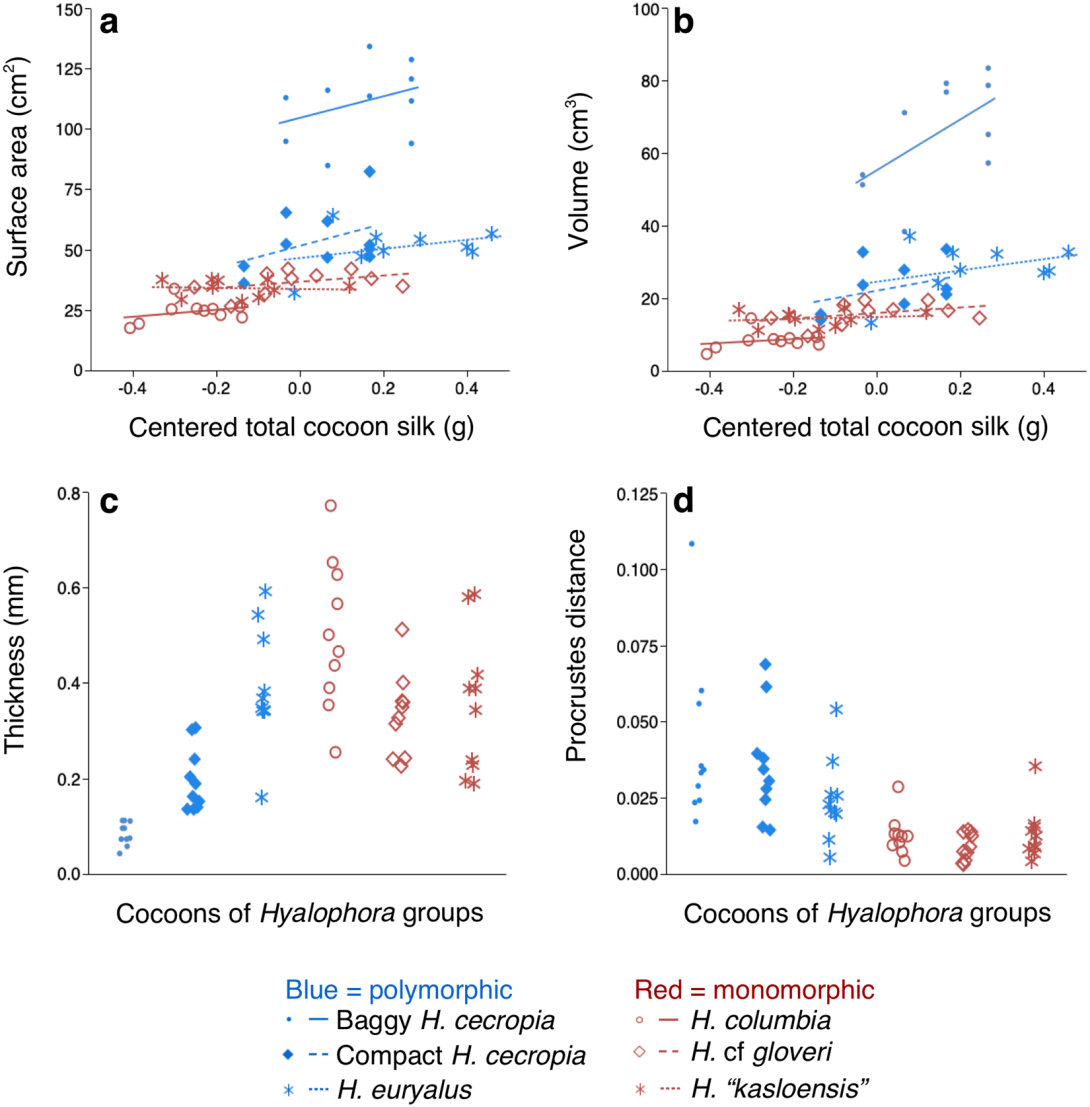
Comparisons of the architectural features of the outer envelopes of cocoons from the different *Hyalophora* groups that we examined. **(a)** Surface area. **(b)** Volume. **(c)** Thickness. **(d)** Shape (as measured using Procrustes distance^22,23^). For **(a**–**d)**, data were analyzed using ANCOVA (n = 10 for all groups).

No significant interaction between *Hyalophora* group and total cocoon silk (ANCOVA: F_5, 5)_ = 0.5788, p = 0.7159) was found in our initial comparison of outer envelope surface areas. In our subsequent ANCOVA analysis with the interaction term removed, we found that the surface areas of cocoons were significantly different between the *Hyalophora* groups (F_(5, 5)_ = 75.7264, p < 0.0001; Fig. 3a). Post hoc comparisons (Tukey HSD test, ∝ = 0.05) found that baggy *H. cecropia* cocoons had the greatest outer envelope surface areas, followed by compact *H. cecropia* and *H. euryalus* cocoons; all of which spin polymorphic cocoons (Fig. 3a). The cocoons with the lowest surface areas were *H. “kasloensis”*, *H*. cf *gloveri*, and *H. columbia*. Cocoons from these three species were similar in surface area and all produce monomorphic cocoons (Fig. 3a). A positive relationship between total cocoon silk and outer envelope surface area was also found (F_(1, 1)_ = 5.0187, p = 0.0294).

Similarly, we found no significant interaction between *Hyalophora* group and total cocoon silk (ANCOVA: F_(5, 5)_ = 1.9169, p = 0.1093) when we initially compared outer envelope volumes. In our subsequent analysis with the interaction removed, we found that the outer envelopes differed in volume (F_(5, 5)_ = 54.8507, p < 0.0001; Fig. 3b). Baggy *H. cecropia* cocoons had outer envelopes with the greatest volume relative to all other cocoons (post hoc Tukey HSD test, ∝ = 0.05; Fig. 3b). Cocoons from all of the other *Hyalophora* groups had similar outer envelope volumes (Fig. 3b), and a positive relationship between total cocoon silk and outer envelope volume was observed (F_(1, 1)_ = 7.2323, p = 0.0096).

In comparing outer envelope thicknesses, we also found no significant interaction between *Hyalophora* group and total cocoon silk (ANCOVA: F_(5, 5)_ = 1.0996, p = 0.3733). Without the interaction term in our analysis, we found that cocoons from the different groups differed in outer envelope thickness (F_(5, 5)_ = 16.5639, p < 0.0001; Fig. 3c), but thickness was not related to the type of cocoon spun (Post hoc Tukey HSD test, ∝ = 0.05; Fig. 3c), nor was thickness related to total cocoon silk (F_(1, 1)_ = 2.6057, p = 0.1125).

In our initial comparison of outer envelope shape, there was no significant interaction between total cocoon silk and *Hyalophora* group (ANCOVA: F_(5, 5)_ = 0.7988, p = 0.5561). In our succeeding analysis with the interaction term omitted, we found that the outer envelopes differed in shape (F_(5, 5)_ = 5.5116, p = 0.0004; Fig. 3d) between groups. The outer envelopes segregated into the following groups according to overall shared shape: *H. euryalus* and *H. columbia* cocoons had similar shape; the rest of the *Hyalophora* groups each had outer envelopes with their own, different shape (post hoc Tukey HSD test, ∝ = 0.05). No relationship between total cocoon silk and outer envelope shape was found (F_(1, 1)_ = 0.2110, p = 0.6479).

### Intermediate space volumes

**Figure 4:**
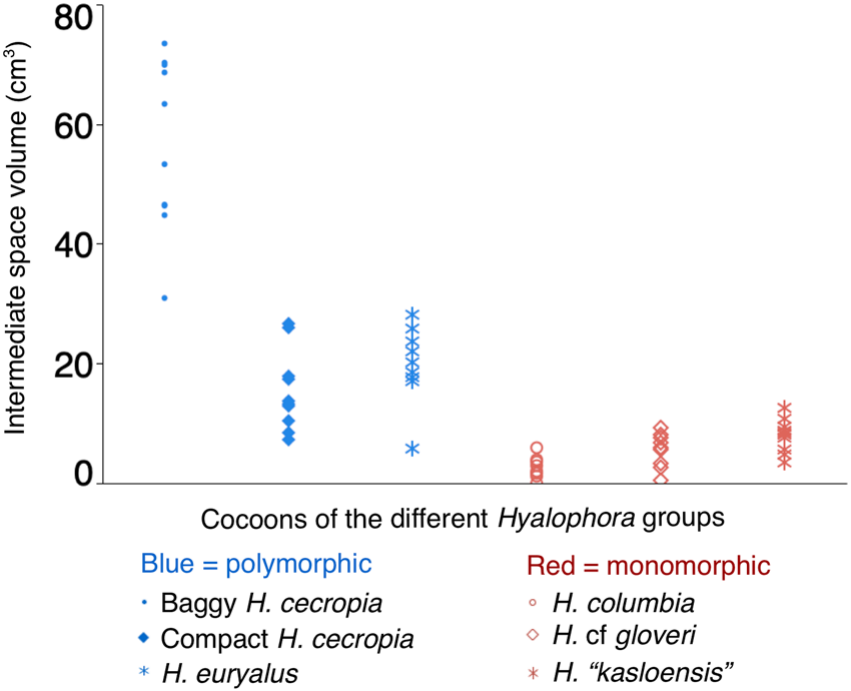
Comparison of the intermediate space volume of cocoons from the different *Hyalophora* groups observed in our study. Data were analyzed using the Kruskal-Wallis test (n = 10 for all groups, except for *H. euryalus* in which n = 9).

We omitted from our analysis a single *H. euryalus* cocoon that had no inner envelope. For one *H. columbia* cocoon, the intermediate space volume could not be measured, as the two distinct envelope layers were spun so close together; this cocoon received a value of zero for intermediate space volume. We found that the intermediate space volume was significantly different between the *Hyalophora* groups (Kruskal-Wallis test: χ^2^ (5) = 47.8515, p < 0.0001; Fig. 4). Cocoons from groups that can spin polymorphic cocoons had the largest intermediate spaces: baggy *H. cecropia* cocoons had the largest intermediate spaces of all cocoons; *H. euryalus* and compact *H. cecropia* cocoons were similar, and had the next largest intermediate space volumes (post hoc Wilcoxon pairwise comparisons, ∝ = 0.05). In contrast, the groups that spin monomorphic cocoons had the smallest intermediate spaces (Fig. 4). *Hyalophora “kasloensis”* and *H*. cf *gloveri* cocoons had similar, low intermediate space volumes, while *H. columbia* cocoons had the smallest intermediate space volumes of all groups.

### Inner envelopes – size and shape

In these comparisons, we omitted the *H. euraylus* cocoon that was without an inner envelope. We also omitted the *H. columbia* cocoon of which it was impossible to separate the inner envelope from the outer envelope properly. Here, the two distinct envelope layers were spun so close together that removal of the inner envelope resulted in an inner envelope layer that was too torn for 3D scanning.

**Figure 5:**
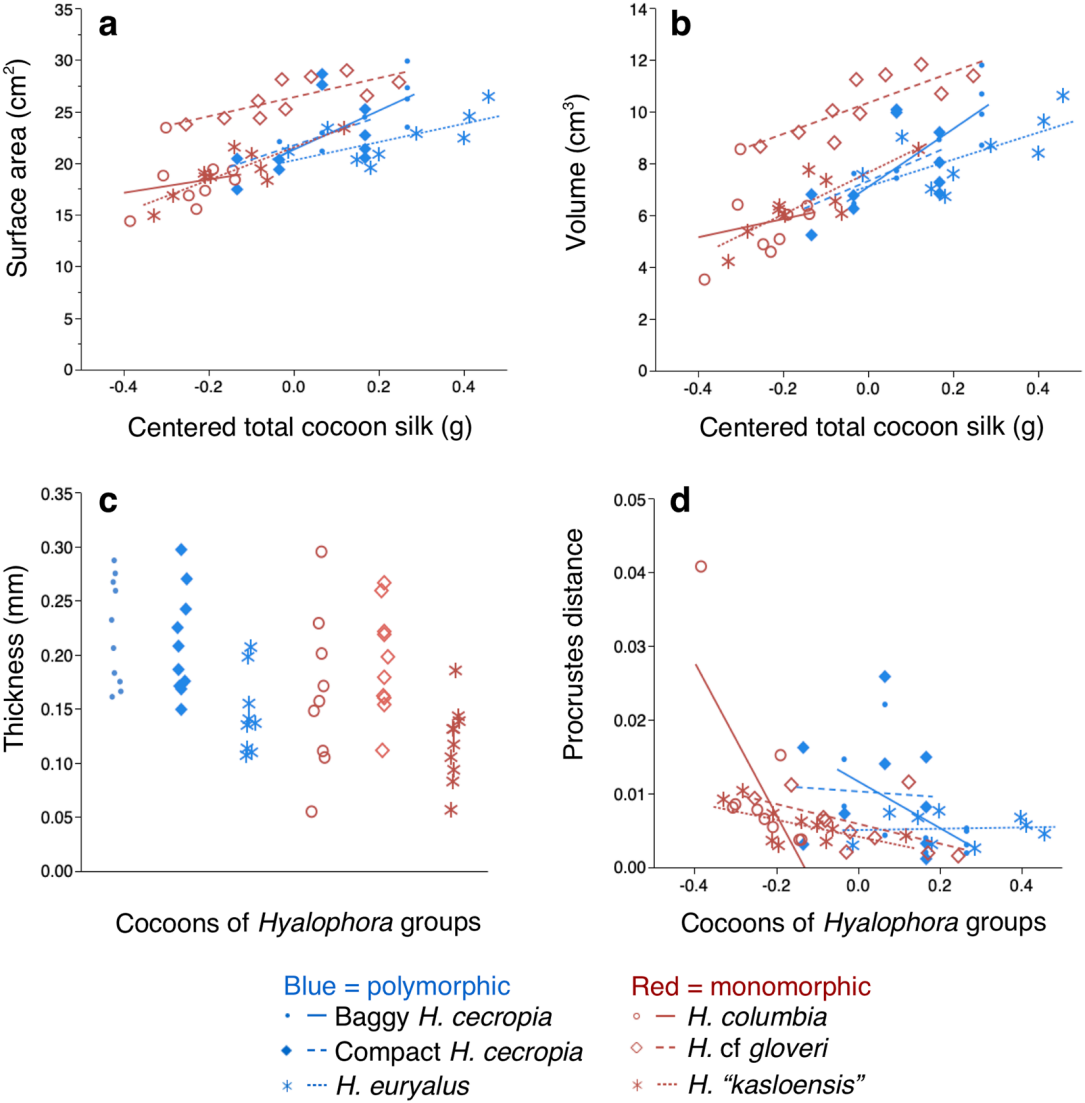
Comparison of the architectural features of the inner envelopes of cocoons from the different *Hyalophora* groups that we examined. **(a)** Surface area. **(b)** Volume. **(c)** Thickness. **(d)** Shape (as measured using Procrustes distance^22,23^). For **(a**–**d)**, data were analyzed using ANCOVA (n = 10 for all groups, except for *H. euryalus* and *H. columbia* in which n = 9).

No interaction between *Hyalophora* group and total cocoon silk was observed when we compared the inner envelopes of the *Hyalophora* groups in both surface area (ANCOVA: F_(5, 5)_ = 0.5817, p = 0.7138) and volume (ANCOVA: F_(5, 5)_ = 0.6839, p = 0.6379). Our subsequent analyses with the interaction term removed showed that the inner envelopes were different among the *Hyalophora* groups in both surface area (F_(5, 5)_ = 11.4633, p < 0.0001; Fig. 5a) and volume (F_(5, 5)_ = 13.6788, p < 0.0001; Fig. 5b). *Hyalophora c. gloveri* inner envelopes had both the greatest surface area and volume of all groups, and all other groups had similar inner envelope surface areas and volumes (post hoc Tukey HSD test, ∝ = 0.05; Fig. 5a-b). Total cocoon silk had a positive relationship with both inner envelope surface area (F_(1, 1)_ = 28.6158, p < 0.0001) and volume (F_(1, 1)_ = 37.2155, p < 0.0001).

An initial comparison of inner envelope thicknesses found no interaction between *Hyalophora* group and total cocoon silk (ANCOVA: F_(5, 5)_ = 1.7775, p = 0.1363). We found that the inner envelopes differed in thickness (F_(5, 5)_ = 7.7078, p < 0.0001; Fig. 5c) in our subsequent analysis that omitted the interaction term, but inner envelope thickness was not related to either cocoon-type (baggy or compact) or the number of cocoon-types that could be spun (polymorphic or monomorphic) (post hoc Tukey HSD test, ∝ = 0.05; Fig. 5c).

When we compared the shapes of the inner envelopes of the different *Hyalophora* groups, we found a significant interaction between *Hyalophora* group and total cocoon silk (ANCOVA: F_(5, 5)_ = 3.2718, p = 0.0131; Fig. 5d). This result shows that the shapes of the inner envelopes of the *Hyalophora* groups will be different for inner envelopes at different sizes.

### Evolution of cocoon morphology

**Figure 6:**
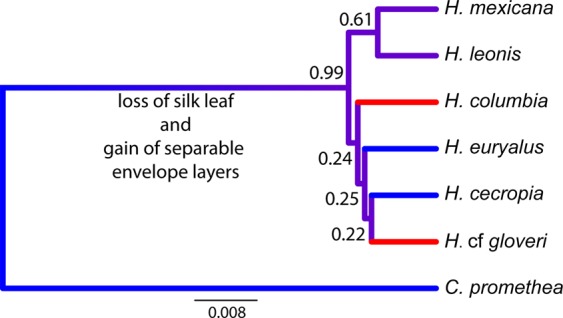
Phylogenetic species tree of *Hyalophora* silk moth species with *C. promethea* outgroup. This tree represents individuals from previous published trees^12,13^, with all hybrid and potentially misidentified individuals removed. Posterior probabilities are indicated at each node, highlighting uncertainty of branching order amongst the rapidly diverging *Hyalophora* lineages. Blue branches indicate species constructing polymorphic cocoons and red branches indicate species that construct monomorphic, compact cocoons only. Purple branches indicate that the presence or absence of polymorphism is unknown (*H. mexicana* and *H. leonis*).

The species tree generated by combining available COI datasets within this group, and removing all proposed hybrids or misidentified individuals, matches previous reconstructions for this clade^12,13^. In particular, this species tree highlights a clear divergence from the outgroup followed by a potential burst of species in *Hyalophora* lineages (characterized by low posterior probabilities at each of the nodes and short branch lengths; Fig. 6). The limitations of the small COI dataset, and the apparent rapid diversification within *Hyalophora*, put the exact character evolution of cocoons within *Hyalophora* beyond the scope of the current work, however. Nevertheless, our analysis demonstrates that *Hyalophora* has diverged from its outgroup (*C. promethea*) as cocoon construction in *Hyalophora* has evolved to include the production of separable, distinct envelopes, and the loss of a silk leaf (which attaches the cocoon to a branch). These traits, and the addition of silk to the intermediate space between envelopes in some of the *Hyalophora* lineages, are not found in the sister clade. Although little is known about cocoons of the newly described *H. mexicana*, and *H. leonis* lineages, the existence of cocoon polymorphism in the outgroup as well as in multiple ingroup species, suggests that cocoon polymorphism is an ancestral state. All *Hyalophora* monomorphic species constructed cocoons with compact morphology, though the uncertainty in phylogenetic relationships limits the interpretation of whether or not this has resulted from a single or multiple loses of polymorphic cocoon shape.

## Discussion

Within *Hyalophora*, we found that all species have evolved cocoons with separable, distinct envelopes, i.e., the outer and inner envelopes. Overall, we found that the cocoons spun by the different *Hyalophora* groups were architecturally different at the level of the outer envelope. This architectural difference in cocoons was manifested as differences in outer envelope size (i.e., surface area and volume) and in intermediate space volume, a cocoon feature directly related to the size of the outer envelope. Between species, the volume of this intermediate space, and the silk contained within it, also varies. For example, *H. cecropia* has a high degree of layer separation compared to other species such as *H. columbia*. The larger separation between the two envelopes has led to the construction of cocoons with significantly larger intermediate spaces and silk. We also found that groups that produce polymorphic cocoons (*H. cecropia* and *H. euryalus*) possess larger outer envelopes than groups that produce monomorphic, compact cocoons (*H. columbia*, *H*. cf *gloveri*, and *H. “kasloensis”*). The larger outer envelopes of groups that can construct polymorphic cocoons appears to be correlated with the amount of total cocoon silk produced, as these groups have cocoons that contain the most total silk.

In contrast to the relationships between species when outer envelopes are compared, the inner envelopes of the different *Hyalophora* groups were similar overall. Any differences that we found between groups had no clear relationship with either architectural features or with cocoon polymorphism. The similar inner envelopes of the different *Hyalophora* groups suggest that these inner envelopes share in the same function, i.e., housing for the individual during pupal to adult development, and are comparable in their biophysical characteristics, presumably in the same manner as to how the inner envelopes of baggy and compact *H. cecropia* cocoons are equivalent in biophysical properties (e.g., thermoregulation and moisture permeability^6^). As separable outer and inner envelopes are a derived trait in *Hyalophora* (Fig. 6), the differences seen between outer envelopes but not in inner envelopes, support the idea that these species have evolved the outer envelope as a key way to help buffer against certain environmental stressors that might be stronger or more prevalent in the habitat range of a particular species^6^ (see below).

In addition to differences in the surface area, volume, and thickness of the outer envelopes of *Hyalophora* cocoons, we also found that outer envelope polymorphism was different across species. Interestingly, cocoon architectural monomorphism, at the level of the outer envelope, is a derived trait in *Hyalophora* (Fig. 6). Two species have lost cocoon polymorphism (*H. columbia* and *H*. cf *gloveri*), with the compact form persisting, potentially in response to environmental conditions.

Previous work with cocoons of *H. cecropia* has demonstrated that compact cocoons are more hydrophobic than baggy cocoons, as they absorbed significantly less water in water absorption trials^6^. This greater level of hydrophobicity is due in part to compact cocoons having significantly thicker outer envelopes than baggy cocoons, with this greater thickness reducing envelope porosity. Moreover, less water is held by compact cocoons due to having smaller intermediate spaces for water to be contained within them^6^. We found that *H. columbia* and *H*. cf *gloveri* had cocoons with significantly thicker outer envelopes than compact *H. cecropia* cocoons. Similarly, *H. columbia* inner envelopes are thicker than the inner envelopes of compact *H. cecropia*, and the inner envelopes of *H*. cf *gloveri* are similar in thickness to that of compact *H. cecropia*. In addition, the intermediate spaces of both *H. columbia* and *H*. cf *gloveri* are significantly smaller than that of compact *H. cecropia* cocoons. Taken together, our results suggest that the thicker envelopes and the more tightly woven architecture of cocoons in both *H. columbia* and *H*. cf *gloveri*, can make these compact cocoons substantially more hydrophobic and less absorptive of water.

We speculate that these enhanced physical barriers to water penetration possessed by these cocoons is consistent with an architectural strategy of freeze avoidance and protection against ice, by which specific architectural features help prevent inoculative freezing and lethal intracellular freezing of the individual^5,14^. Reducing freezing risk and exposure to external ice is particularly important during periods of pupal development during which rain and subzero temperatures can coincide, leading to harmful ice formation such as on or within the pupa^15^. This function has been observed in the cocoons used for overwintering in other insect species^16,17^. Freezing, facilitated by exposure to water, is lethal for many insects^18,19^.

**Figure 7:**
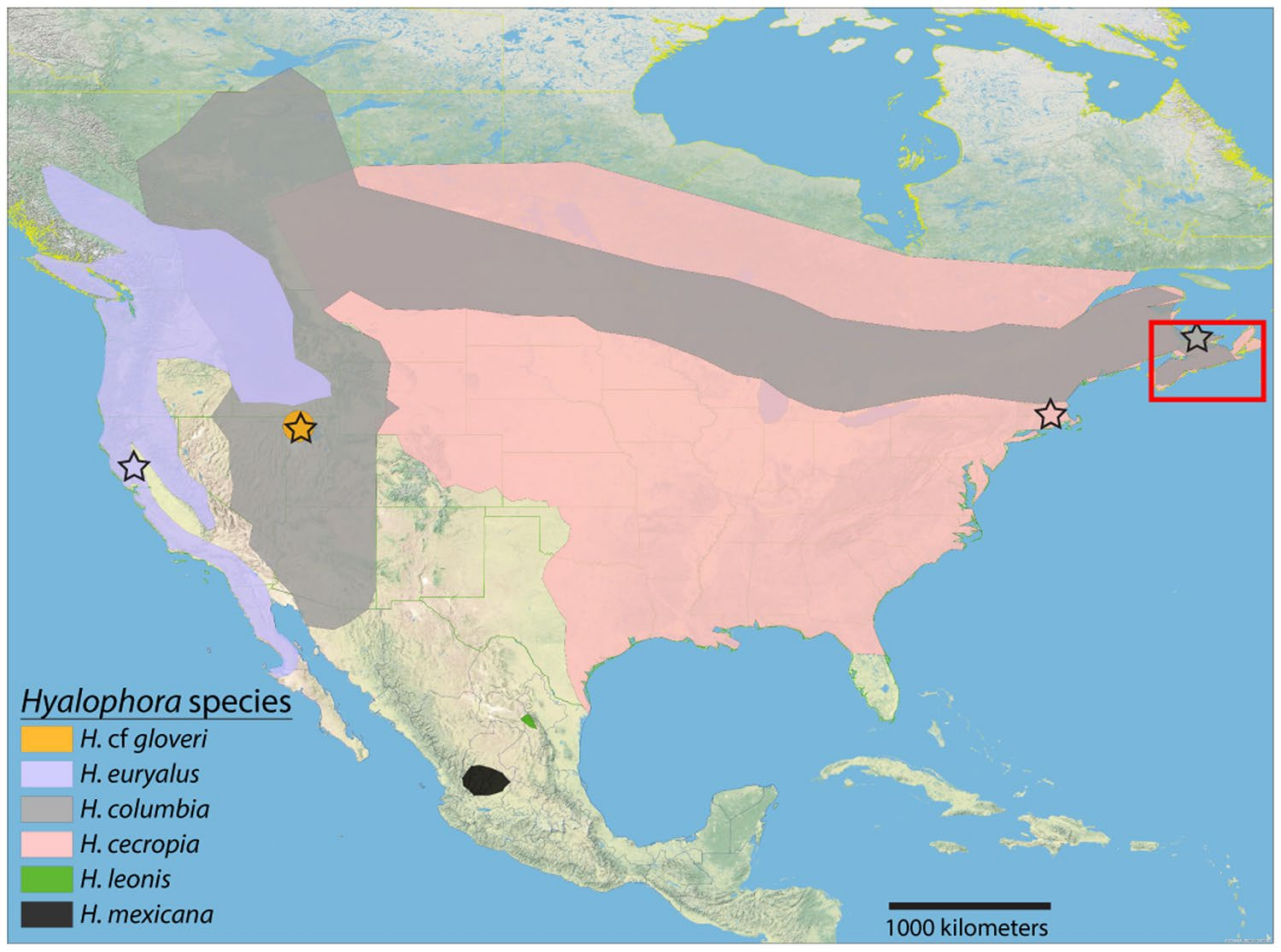
Collection localities and estimated range maps of *Hyalophora* silk moth species in North America. Stars indicate collection locations of cocoons for that species. The area within the red rectangle indicates anecdotal observations where only compact *H. cecropia* cocoons have been found^30^. Range maps were estimated from previous work^8,12,13^, and collection localities logged in www.butterfliesandmoths.org. The ranges of the two subspecies of *H. columbia* (*H. c. columbia* and *H. c. gloveri*) are merged here due to paraphyly found in genetic reconstructions. As the ranges of the newly identified species of *H. mexicana* and *H. leonis* are still unsure, these areas may significantly underrepresent the actual ranges. Likewise, the actual range of *H*. cf *gloveri* is also uncertain except for a small general region in Utah near Salt Lake City that is the only known locality of this distinct genetic lineage. Map was created using ESRI ArcGIS software, version 10.4 (https://www.esri.com), and cocoon collection locations were added onto the map using Adobe Illustrator CS6, version 16.0.2 (https://www.adobe.com).

For example, although found in similar ranges as that of *H. cecropia* and *H. euryalus*, overall, the range of *H. columbia* in North America is limited to areas that typically are more temperate and that can experience much colder seasonal temperatures. The architecture of *H. columbia* cocoons, (i.e., all compact, with accentuated architectural features of the compact morph including significantly thicker envelopes and more tightly woven structure) might result from directional selection for the compact morph in response to the more probable environmental stress that occurs in these areas (e.g., cold temperatures leading to freezing). Cocoons of *H*. cf *gloveri* might have experienced similar directional selection that has caused this species to produce monomorphic, compact cocoons. Consistent with this hypothesis is that the construction of baggy and compact cocoons in *H. cecropia* appears to vary with location under natural conditions. Anecdotal observations of cocoons in more northern and colder habitats that can experience intense fall and winter storms with heavy rain, ice, and cold temperatures (e.g., Nor’easters), found that all cocoons possessed compact cocoon morphology (e.g., Nova Scotia, Canada – Fig. 7, area within red rectangle; Ferguson 1972). In addition, observations of cocoons in the St. Louis, Missouri area (1910–1:4 baggy:compact, 1911–1:6 baggy:compact^20^), and of cocoons in Urbana-Champaign, Illinois (1965–1966–1:6 baggy:compact^21^), found that cocoons had a greater probability of being compact.

Cocoon construction in *Hyalophora* silk moths represents a useful model to examine the evolution of diversity in animal architecture, especially in closely related groups. For example, our current study can allow us to make predictions on the cocoon architecture of other *Hyalophora* species (e.g., *H. leonis* and *H. mexicana*, which might have monomorphic compact cocoons given their limited ranges and mountainous distributions similar to that of *H*. cf *gloveri*; Fig. 7) and that of other cocoon-making insects not directly studied here. Moreover, due to contemporary stressors such as climate change, which bring a greater probability of extreme weather events and increased environmental stochasticity, cocoon construction will likely be under intense selection pressure. Such stress might potentially select for the rapid evolution of the architectural characteristics of cocoons. Further genetic data and functional experiments on cocoons are now needed to better understand the evolution of cocoon architecture within *Hyalophora* and quantify their effects on fitness and survivorship.

## Methods

### Cocoons

We compared the architectural features of cocoons that were spun by several species of *Hyalophora* silk moth (*H. cecropia*, *H. columbia*, *H*. cf *gloveri*, and *H. euryalus*), and that of the hybrid *H. “kasloensis”*. The cocoons from each of these groups (n = 10 cocoons for each group) were all collected from various locations in the United States (Fig. 7). For *H. cecropia*, which has two discrete cocoon morphs (baggy and compact), we sampled both types of cocoons (10 baggy and 10 compact) from Central and Eastern Massachusetts that were previously characterized^6^ (cocoons collected by Steven M. Reppert). *Hyalophora euryalus* produces cocoons with continuous polymorphic architectural morphology ranging from compact to baggy construction throughout its range^8,9^. Samples in our study consisted of *H. euryalus* cocoons collected in Lake County, California (38°38′02.2776′′N, −122°36′04.5360W; collected by Scott Smith) that were looser and rounder than those found in other portions of their range^8,9^. The other *Hyalophora* groups that we examined all produce monomorphic compact cocoons: compact *H. columbia* cocoons from Prince Edward Island, Canada (46°30′38.5632, −63°25′0.5304′′; collected by Scott Smith); compact *H*. cf *gloveri* cocoons from Sandy City, Utah (40°34′21.00′′, −111°51′34.9992′′; collected by Scott Smith); and compact *H. “kasloensis”* cocoons similar to *H*. cf *gloveri*^8^ from Spokane, Washington (47°39′ 31.6087′′, −117°25′33.7678′′; collected by Scott Smith). The closely related silk moth *Callosamia promethea* was used as the outgroup in our phylogenetic analysis, as based on previous work^12^ (see below). This species produces both baggy and compact cocoon morphs^8^. Compact *C. promethea* cocoons collected in Eastern Massachusetts (collected by Steven M. Reppert in the same areas *H. cecropia* cocoons were collected^6^) were used for morphological comparisons (n = 10 cocoons; see Supplementary Fig. S1).

### Silk building materials

For each cocoon from the different *Hyalophora* species, we separately weighed the amounts of silk that formed the outer envelope, any silk that was contained within the intermediate space, and the silk that formed the inner envelope on a Mettler-Toledo balance (model ME204E). We calculated the total silk mass for each cocoon by adding these three silk masses together. We then determined the percentage of total cocoon silk that was contained in each of these three different cocoon sections for each cocoon. We compared the total amount of cocoon silk and the percentage of total cocoon silk that was allocated to each of the three different cocoon sections (i.e., the outer envelope, the intermediate space, and the inner envelope) across the different *Hyalophora* groups.

### 3D cocoon scanning

In order to compare the architectural properties of the different cocoon sections (outer envelope, intermediate space, and inner envelope) from the different *Hyalophora* groups that we collected, we quantified the architectural features of cocoons using previously published methods^6^. For all cocoons, we removed any leaves or branches that remained attached to a cocoon after the cocoon was extracted from its outdoor collection site. We then produced 3D scans of each cocoon using a MakerBot Digitizer 3D scanner (MakerBot Industries). For each cocoon, an initial scan of the whole cocoon was made to obtain an outer envelope scan. We then obtained an inner envelope scan by removing the outer envelope and any silk contained within the intermediate space, following the scanning of the whole cocoon.

### Cocoon size

To produce measurements that assess cocoon size, we obtained surface area and volume measurements for both the outer and inner envelopes of each cocoon by importing each 3D scan into the program netfabb Basic (Version 5.2.0, Autodesk, Inc.)^6^. We then obtained the volume of the intermediate space of each cocoon by subtracting the volume of the inner envelope from the volume of the outer envelope. As another measure of cocoon size, we obtained thickness measurements for the outer and inner envelopes of each cocoon by measuring each envelope at its intersection of the vertical and horizontal midline points^6^ using digital calipers (iGaging Precision Instruments). We compared the surface area, volume, and thickness of the different envelope types (baggy or compact; outer or inner), and the volume of the intermediate space, across the different *Hyalophora* groups.

### Cocoon shape

We compared cocoon shapes using methods similar to those used previously^22^ for comparing shapes. We first imported the 3D scans for both envelope types of each cocoon into the 3D visualizing program Amira (Version 6.0.1, Thermo Fisher Scientific). Using Amira, we positioned six separate homologous 3D landmarks on each cocoon (both outer and inner envelopes of each cocoon) to obtain 3D morphometric data (XYZ coordinates) that described the shape of each envelope-type. Homologous points were determined along the long axis of the cocoon, based on the position of the exit valve (top of cocoon) and the center of the base (bottom of cocoon) of cocoons. The 3D morphometric data for the outer and inner envelopes of each cocoon were then analyzed in the morphometric analysis program MorphoJ^23^. We obtained a Procrustes distance value for each of the outer and inner envelopes of each cocoon. Procrustes distance is a metric that allows for the standardization of different objects in order to compare their shapes, without the effects of size (e.g., size differences between objects) affecting the comparisons to be made. We used Procrustes distance as an overall measure of shape. In two separate analyses, we determined if each of the outer and inner envelopes of the different *Hyalophora* groups significantly differed in shape across groups.

### Statistical analyses

Prior to statistical analyses, we checked if our data were normally distributed. Normally distributed data were analyzed using parametric tests. For data that were non-normally distributed, we used appropriate data transformations (arcsine square-root transformation for proportion data and log transformation for all other data types) and re-checked for normality. Data that were normally distributed after transformation were analyzed with parametric tests using the transformed data; for data that remained non-normally distributed even after transformation, we analyzed the untransformed data using non-parametric tests.

Total cocoon silk data were normally distributed, and we analyzed these data using a parametric one-way ANOVA, followed by a post hoc Tukey HSD test. Data examining the percentage of silk that was allocated to either the outer envelope, the intermediate space of cocoons, or to the inner envelope, were each analyzed using non-parametric Kruskal-Wallis tests, followed by a post hoc Wilcoxon pairwise comparison analysis. Intermediate space volume data were also non-normally distributed, and these data were analyzed using these same non-parametric tests. We found significant differences in the amount of total silk in cocoons across groups (see below), which might influence the architectural parameters of cocoons, i.e., surface area, volume, envelope thickness, and shape. We therefore examined the outer envelopes in each of these four architectural parameters using separate parametric one-way ANCOVAs with the amount of total cocoon silk as a covariate (the covariate was centered prior to analysis). ANCOVAs were rerun with the interaction term removed from the analysis, when no significant interaction between *Hyalophora* group and total cocoon silk was found, indicating that all *Hyalophora* groups shared the same relationship with the covariate (i.e., same slope). We followed each ANCOVA analysis with a post hoc Tukey HSD test. To compare inner envelopes across the *Hyalophora* groups in the four architectural parameters, we performed parametric ANCOVAs and post hoc Tukey HSD tests in the same manner as we did with our outer envelope analyses. We performed all statistical analyses in JMP Pro (Version 12.1.0, SAS Institute Inc.).

### Phylogenetic analysis of cocoon architecture

In order to create a more robust species tree to understand cocoon diversification in *Hyalophora*, we first compiled a COI barcode dataset from previously published sources^12,13^, along with samples from the BOLD Systems barcode database (boldsystems.org), resulting in a dataset of 84 individuals which included a single *C. promethea* as the outgroup. All individuals that were indicated as possible “hybrids” in these studies were removed from further analyses (see Supplementary data online). Due to strong monophyly of major clades found in previous work^12,13^, and potential uncertainty of identification culled from the barcode database, we first assessed phylogenetic relationships of all individuals in BEAST v. 1.10^24^. Due to the close relationship and recent divergences expected within the analysis, a strict clock and a constant population size coalescent tree prior were used along with a codon-based SRD06 nucleotide substitution model^25^. The simulation was run for 10 million generations with sampling every 1,000 generations, with the first 10% discarded as burnin. Maximum clade credibility trees were calculated in TreeAnnotator from the Beast package. We assessed the performance of all Bayesian analyses (convergence and stationarity) with the program Tracer v. 1.5^26^. Only runs with adequate mixing and an Effective Sample Size (ESS) above 200 were considered for final analyses.

In order to correctly assign species identity for the SpeciesTree analysis, we first assessed the groupings of each specimen to remove those that were potentially misidentified. Three individuals from the barcode database that had unexpected phylogenetic relationships based on their taxonomic IDs, were subsequently removed from further analyses. Based on previous findings^12,13^, which showed paraphyly between *H. c. gloveri* “a” and *H. c. columbia*, these two subspecies were collapsed into the *H. columbia* lineage. The other monophyletic lineages included in the species tree were *H. euryalus*, *H. c. gloveri* “b” (referred to as *H*. cf *gloveri* onward, all collected from Utah), *H. mexicana*, and *H. leonis*^12,13^.

To generate the species tree, we used the multispecies coalescent model implemented in StarBEAST2^27^ in BEAST 2.4.8^28^. All individuals were assigned to these four taxon sets along with the *C. promethea* outgroup. We ran StarBeast2 for 100 million generations with 10% burnin, sampling every 5000. Runs were assessed for adequate mixing and ESS above 200 in Tracer as above. A maximum clade creditability tree was created in TreeAnnotator (part of the BEAST package). Additionally, all trees were visualized with DensiTree v. 2.2.5^29^ to observe concordance across trees. Due to low resolution in branching order within *Hyalophora*, ancestral state reconstructions within *Hyalophora* could not be performed and interpretations are limited to comparisons of ingroup and outgroup traits.

## Supplementary information

Supplementary Figure S1.

Supplementary Data.

## Data Availability

All data generated or analyzed during this study are included in this article and its Supplementary files that are online.
