# Supplementary material for: Architectural evolution in cocoons spun by Hyalophora (Lepidoptera; Saturniidae) silk moth species
Source: Sci Rep. 2020 Mar 27;10:5615. doi: 10.1038/s41598-020-62547-1 (PMC7101368; doi:10.1038/s41598-020-62547-1)
Supplement: Supplementary file 1 — Supplementary Figure S1. [file 41598_2020_62547_MOESM1_ESM.pdf]

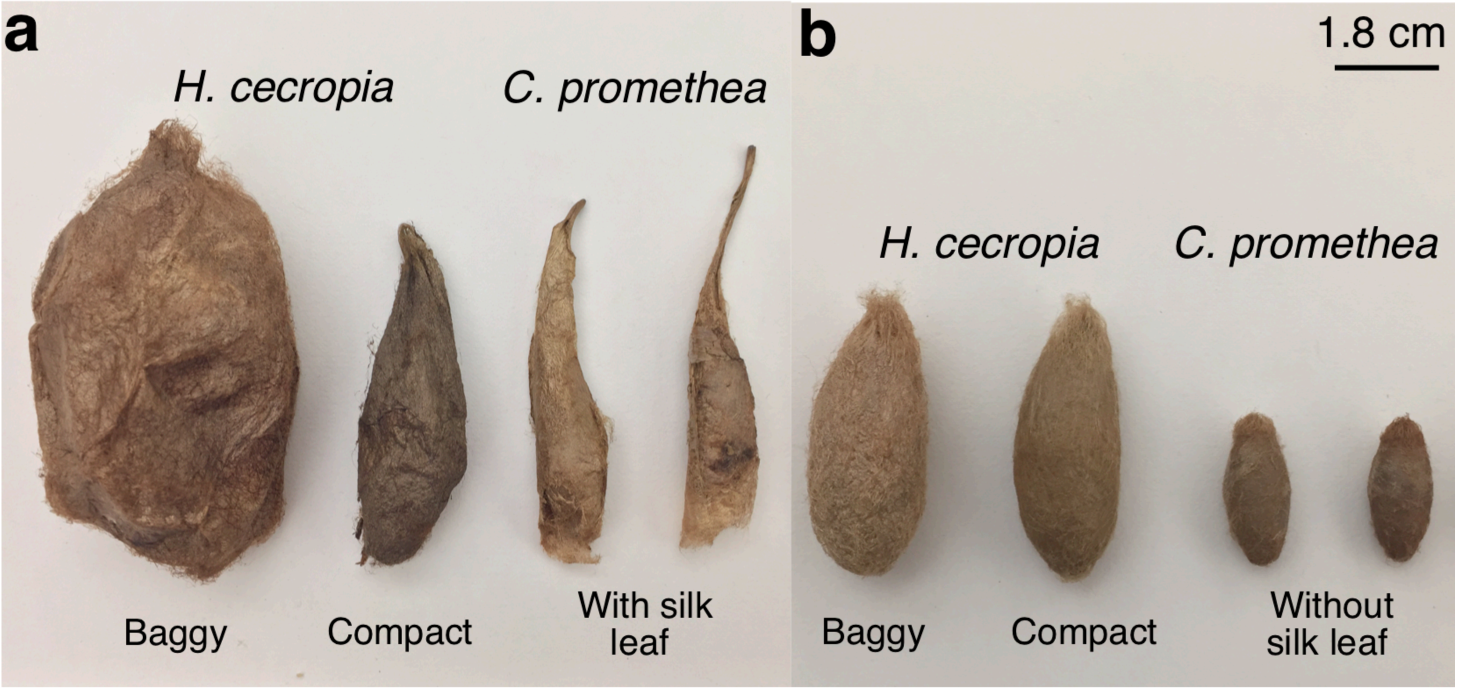

Supplementary Figure S1. Cocoons of one of the *Hyalophora* species as compared with the outgroup species, *Callosamia promethea*. (a) Comparison of cocoons made by one of the species in *Hyalophora* that we examined (baggy and compact *H. cecropia* cocoons) with cocoons representative of those made by *C. promethea*, the outgroup species in our phylogenetic analysis. *Callosamia promethea* cocoons are pictured with their silk leaf attachment still in place (a), whereas these same cocoons (b) have had the silk leaf attachment removed thereby exposing the cocoon.
