# Supplementary material for: Architectural evolution in cocoons spun by Hyalophora (Lepidoptera; Saturniidae) silk moth species
Source: Sci Rep. 2020 Mar 27;10:5615. doi: 10.1038/s41598-020-62547-1 (PMC7101368; doi:10.1038/s41598-020-62547-1)
Supplement: Supplementary file 2 — Supplementary Data. [file 41598_2020_62547_MOESM2_ESM.pdf]

Supplementary Data. List of compiled COI barcode data used in phylogenetic analysis.

Genbank ID and initial proposed species	species assignment following initial phylogenetic reconstructions.
AB015868_Hyalophora_cecropia	Hyalophora_cecropia
AY165720_Hyalophora_cecropia	Hyalophora_cecropia
GU702999_H_gloveri_a	Hyalophora_columbia
GU703000_H_euryalus_kasloensis	Hyalophora_euryalus
GU703001_H_euryalus	Hyalophora_euryalus
GU703002_H_euryalus	Hyalophora_euryalus
GU703009_H_mexicana_PT	Hyalophora_mexicana
GU703460_H_mexicana_PT	Hyalophora_mexicana
GU703461_H_mexicana_HT	Hyalophora_mexicana
GU703462_H_mexicana_PT	Hyalophora_mexicana
GU703463_H_cecropia	Hyalophora_cecropia
GU703464_H_columbia_a	Hyalophora_columbia
GU703465_H_columbia_a	Hyalophora_columbia
GU703533_H_gloveri_b	Hyalophora_gloveri_b
GU703534_H_gloveri_b	Hyalophora_gloveri_b
GU703535_H_euryalus	Hyalophora_euryalus
GU703536_H_euryalus	Hyalophora_euryalus
GWNC613_07_Hyalophora_gloveri_Alberta	Hyalophora_columbia
HM383529_H_columbia	Hyalophora_columbia
HM867376_Hyalophora_euryalus	Hyalophora_euryalus
HM867407_Hyalophora_euryalus	Hyalophora_euryalus
HQ579813_C_promethea	Callosamia_promethea
HQ579814_H_columbia	Hyalophora_columbia
HQ579815_H_columbia_a	Hyalophora_columbia
HQ579817_H_gloveri_a	Hyalophora_columbia
HQ579819_H_cecropia	Hyalophora_cecropia
KJ865741_Hyalophora_gloveri	Hyalophora_columbia

KJ865742_Hyalophora_gloveri	Hyalophora_columbia
KJ865743_Hyalophora_gloveri	Hyalophora_columbia
KJ865744_Hyalophora_gloveri	Hyalophora_columbia
KJ865745_Hyalophora_gloveri	Hyalophora_columbia
KJ865746_Hyalophora_gloveri	Hyalophora_columbia
KM287183_Hyalophora_cecropia	Hyalophora_cecropia
KM287184_Hyalophora_gloveri	Hyalophora_columbia
KM287185_Hyalophora_cecropia	Hyalophora_cecropia
KM287188_Hyalophora_euryalus	Hyalophora_euryalus
KM287189_Hyalophora_gloveri	Hyalophora_gloveri_b
KM287190_Hyalophora_cecropia	Hyalophora_cecropia
KM287191_Hyalophora_mexicana	Hyalophora_mexicana
KM287193_Hyalophora_cecropia	Hyalophora_cecropia
KM287194_Hyalophora_gloveri	Hyalophora_columbia
KM287195_Hyalophora_cecropia	Hyalophora_cecropia
KM544952_Hyalophora_cecropia	Hyalophora_cecropia
KM995807_Hyalophora_leonis	Hyalophora_leonis
KM995808_Hyalophora_sp_columbia_a	Hyalophora_columbia
KM995809_Hyalophora_leonis	Hyalophora_leonis
KM995810_Hyalophora_sp_columbia_a	Hyalophora_columbia
KM995811_Hyalophora_leonis	Hyalophora_leonis
KM995812_Hyalophora_leonis	Hyalophora_leonis
KT130513_Hyalophora_cecropia	Hyalophora_cecropia
KT131868_Hyalophora_columbia	Hyalophora_columbia
KT132640_Hyalophora_cecropia	Hyalophora_cecropia
KT133088_Hyalophora_cecropia	Hyalophora_cecropia
KT137523_Hyalophora_cecropia	Hyalophora_cecropia
KT140564_Hyalophora_cecropia	Hyalophora_cecropia
KT143512_Hyalophora_cecropia	Hyalophora_cecropia
KT147422_Hyalophora_cecropia	Hyalophora_cecropia

KT148193_Hyalophora_cecropia	Hyalophora_cecropia
LBCG332_08_Hyalophora_euryalus_Kamloops	Hyalophora_euryalus
LBCG383_08_Hyalophora_euryalus_Kamloops	Hyalophora_euryalus
LNC911_06_Hyalophora_cecropia_North_Carolina	Hyalophora_cecropia
LOPN217_06_Hyalophora_euryalus_Oregon	Hyalophora_euryalus
LSUSA183_06_Hyalophora_cecropia_Kentucky	Hyalophora_cecropia
LTOL408_07_Hyalophora_gloveri_Utah	Hyalophora_gloveri_b
LTOL278_07_Hyalophora_euryalus_California	Hyalophora_euryalus
LTOL436_07_Hyalophora_cecropia_Maryland	Hyalophora_cecropia
LTOL437_07_Hyalophora_euryalus_California	Hyalophora_euryalus
LTOL438_07_Hyalophora_cecropia_Colorado	Hyalophora_cecropia
RDLQF466_06_Hyalophora_columbia_Quebec	Hyalophora_columbia
RDLQF467_06_Hyalophora_columbia_Quebec	Hyalophora_columbia
RDNMF798_08_Hyalophora_columbia_Ontario	Hyalophora_columbia
RDNMF799_08_Hyalophora_gloveri_Alberta	Hyalophora_columbia
RDNMF800_08_Hyalophora_gloveri_Alberta	Hyalophora_columbia
RDNMF801_08_Hyalophora_gloveri_Alberta	Hyalophora_columbia
RDNMF802_08_Hyalophora_gloveri_Alberta	Hyalophora_columbia
RDNMF803_08_Hyalophora_gloveri_Alberta	Hyalophora_columbia
RDNMF804_08_Hyalophora_gloveri_Alberta	Hyalophora_columbia
RDNMH371_09_Hyalophora_gloveri_Arizona	Hyalophora_columbia
SATOL007_07_Hyalophora_columbia_Michigan	Hyalophora_columbia
SATOL056_07_Hyalophora_cecropia_Wisconsin_captiv	Hyalophora_cecropia
SDHC360_08_Hyalophora_mexicana_Zacatecas	Hyalophora_mexicana
